# Intravascular Glomus Tumor of the Forearm Causing Chronic Pain and Focal Tenderness

**DOI:** 10.1155/2014/619490

**Published:** 2014-02-03

**Authors:** Sang Ki Lee, Dae Geon Song, Won Sik Choy

**Affiliations:** Department of Orthopedic Surgery, College of Medicine, Eulji University, 1306 Dunsan-dong, Seo-gu, Daejeon 302-799, Republic of Korea

## Abstract

*Introduction*. A glomus tumor is a benign vascular tumor derived from glomus cells and occurs mainly in the subcutaneous layer of the subungual or digital pulp. Extradigital glomus tumors have been reported within the palm, wrist, forearm, foot, bone, stomach, colon, cervix, and mesentery. Glomus tumors can originate from the intraosseous, intramuscular, periosteal, intravascular, and intraneural layers. However, a glomus tumor originating from the intravascular layer of the forearm is a rare condition. *Case Report*. A 44-year-old woman had a 7-year history of chronic pain and focal tenderness of the forearm. No hypersensitivity or sensory alterations were observed. Contrast magnetic resonance imaging (MRI) showed a mass measuring 5 × 3 × 2 mm leading to a vein. Surgical excision was performed, and the tumor was completely resected. Finding of gross examination revealed a dark-red, well-defined soft tissue tumor, and histologic examination confirmed that the mass was a glomus tumor. The patient's symptoms were completely resolved postoperatively. *Conclusion*. Intravascular glomus tumors rarely occur in the forearm; therefore, a thorough physical exam, comprehensive medical history, in-depth imaging, and early surgical excision upon clinical suspicion may be helpful to prevent a delayed or incorrect diagnosis.

## 1. Introduction

The normal glomus body is a specialized arteriovenous anastomosis that plays a role in thermoregulation [1]. A glomus tumor is a benign type of vascular neoplasm composed of modified smooth muscle cells and is thought to originate from the glomus body within reticular dermis [2]. Glomus tumors are most frequently found in the extremities, usually on the digits [3]. They are also located on extradigital sites, including the palm, wrist, forearm, foot, bone, stomach, colon, cervix, and mesentery [4]. Various origins of glomus tumors have been reported, including intramuscular, periosteal, intraosseous, intravascular, and intraneural locations [3, 5]. Although the forearm is the most common extradigital location [3], intravascular origination of a forearm glomus tumor is an exceptionally rare occurrence. To the best of our knowledge, only 3 cases of intravascular forearm glomus tumors have been reported [1, 6, 7]. Here, we present a rare case of a patient with chronic pain and focal tenderness caused by an intravascular glomus tumor in the forearm.

## 2. Case Report

A 44-year-old woman presented with a 7-year history of occasional pain on the volar-radial aspect of her right mid-forearm. The patient reported that the pain had gradually worsened and was significantly increased on palpation. She recalled no traumatic event and reported no relevant family history. On physical examination, “spark-like” pain was caused by touching a well-localized area, but no sensory alterations or temperature hypersensitivity was observed. No contour deformities or visible palpable skin lesions were identified.

**Figure 1:**
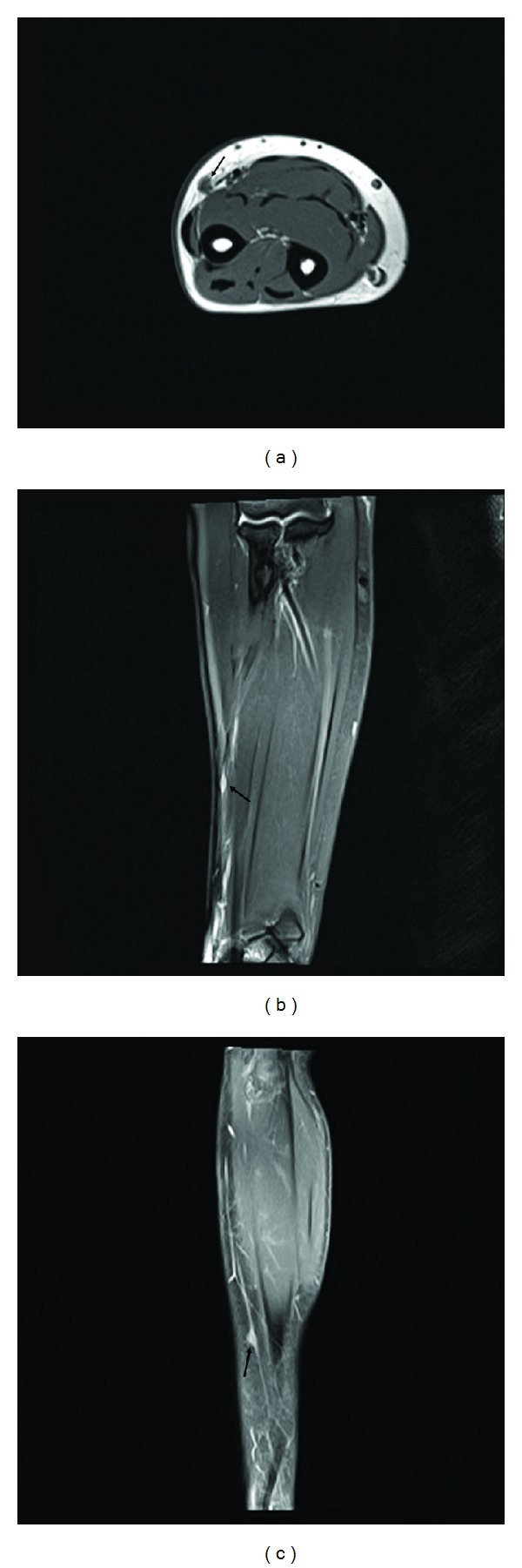
(a) MRI axial T1-weighted image showing an intermediate intensity mass on the subcutaneous layer of the volar-radial aspect of the forearm (arrow). (b) MRI coronal T2-weighted image showing a well-defined high signal mass that is especially prominent in the small vessel (arrow). (c) MRI sagittal image with contrast displaying a homogenous strong enhancement (arrow).

Laboratory examinations, plain radiographs, and computed tomography (CT) revealed normal findings. Magnetic resonance imaging (MRI) with contrast enhancement demonstrated an oval-shaped subcutaneous mass leading to a small vessel. The size of the mass was determined to be 5 × 3 × 2 mm on MRI. The mass had intermediate intensity on T1-weighted images and displayed an inhomogeneous high signal on T2-weighted images and homogenous strong enhancement with contrast (Figure 1).

**Figure 2:**
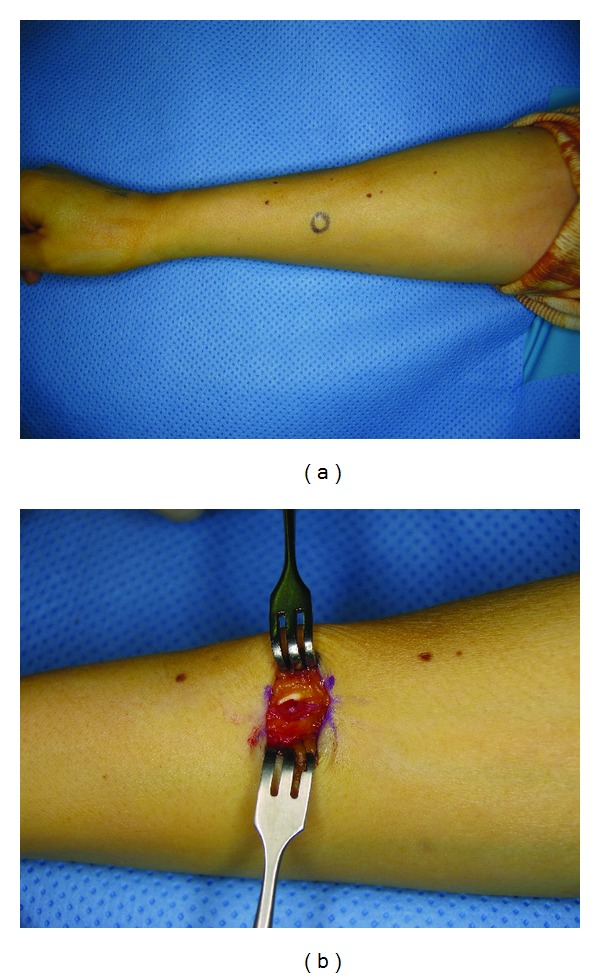
(a) Intraoperative gross photograph showing the focal area of tenderness, which was marked before incision. (b) An oval-shaped, well-encapsulated mass was exposed through careful dissection, and was identified to be lying in a branch of the cephalic vein.

**Figure 3:**
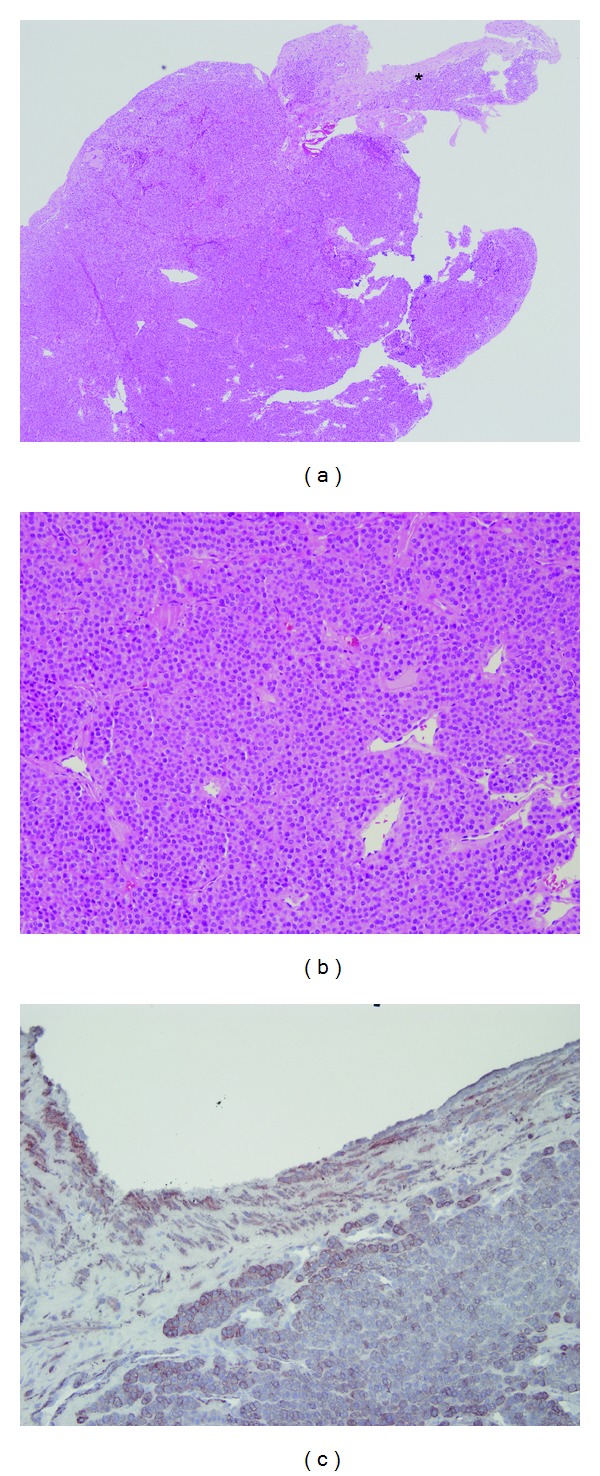
(a) Histopathology slide of the specimen with hematoxylin and eosin staining at 40x magnification shows glomus tumor cells adjacent to the vessel wall (asterisk). (b) The tumor consists of uniformly rounded cells with centrally located round nuclei, eosinophilic cytoplasm, and dilated capillary vessels (200x magnification). (c) The tumor glomus cells and the smooth muscle cells of the vein wall stained for smooth muscle actin.

During the operation, a 3 cm longitudinal incision was made on the radial aspect of the forearm at the site of tenderness. A dark-red, well-defined, and oval-shaped mass (diameter, 3 mm) was identified in a branch of the cephalic vein (Figure 2). The mass was carefully resected from the vascular structure, and the vein was then ligated. Histologic examination confirmed that the mass was a glomus tumor, appearing as uniformly rounded cells with centrally located round nuclei and capillary size vessels surrounding the mass. Peripherally, the tumor was contiguous to the smooth muscle layer of the affected cephalic vein. Immunohistochemical staining revealed that the tumor cells were positive for smooth muscle actin (Figure 3).

After complete surgical excision, the patient's symptoms fully resolved within days. There was no recurrence of pain up to 6 months postoperatively.

## 3. Discussion

The normal glomus body is involved in thermal regulation through control of the skin circulation [3, 8]. It is composed of an afferent arteriole, which is derived from the small arterioles supplying the dermis [4]. Glomus tumors are considered to be a hamartomatous proliferation of modified smooth muscle cells originating from normal glomus cell populations [8]. Glomus tumors account for 1-2% of the soft tissue tumors of the hand and are well recognized by orthopedic surgeons as a painful subcutaneous nodule in a subungual or digital pulp location [9, 10]. Importantly, extradigital cases account for 11–65% of all glomus tumor cases and have been reported to be more common in men, although most subungual lesions occur in women [3, 9, 11]. The etiology of a glomus tumor is not clear, although an autosomal dominant pattern of inheritance and a history of injury have been reported [12, 13]. We present the case of a woman who had no family history of a relevant glomus tumor and no history of injury; chronic pain and focal tenderness were alleviated after surgical removal of an extradigital forearm glomus tumor.

The triad symptoms of a glomus tumor are paroxysmal pain localized tenderness and cold intolerance. A glomus tumor can occasionally cause pain by directly compressing the forearm nerves [14, 15]. However, glomus cells are adjacent to a rich neutral bed; this may explain the severe pain and hypersensitivity associated with these lesions [3]. In addition, mast cell of the glomus tumor, may play an important role in the mediation of pain [16]. As previously mentioned, the forearm is the most common extradigital location [3], and the lesions most commonly occur in the subcutaneous layer, with very infrequent occurrence in extracutaneous locations such as muscle, bone, and blood vessels [1, 6–8, 17]. Superficially located glomus tumors often produce typical symptoms such as paroxysmal pain, exacerbated by changes in temperature [9]. However, glomus tumors located at an unusual site may present with features of no pain or hypersensitivity [7, 8, 17]. Indeed, there was no sensory alteration or temperature hypersensitivity in the current case in which the glomus tumor was located intravascularly.

It is difficult to objectively detect a glomus tumor through clinical palpation because of the subcutaneous or deeper layer location and the small size (within a few millimeters). Imaging studies such as MRI or ultrasonography are useful for diagnosis [2, 10]. MRI in particular can verify the presence of small soft tissue tumors such as extradigital glomus tumors, epidermal cysts, fibromas, synovial cysts, and venous malformations. A review of the literature on MRI findings of extradigital glomus tumors showed that these lesions are oval-shaped and well defined and demonstrate hypo- or isointensity on T1-weighted images and hyperintensity on T2-weighted images [2]. In the present case, T1-weighted MRI images showed an intermediate intensity, and T2-weighted images showed an inhomogeneous high signal. However, in a previous study, MRI failed to reveal any lesion [3], and the main role of these imaging studies is to identify the presence of a lesion. A definitive diagnosis is only established in a histologic examination performed after surgical excision; this examination can identify glomus tumor cells, which are distinct of uniformly rounded cells with centrally located round nuclei.

The only treatment for a glomus tumor is surgical excision. Recurrence is infrequent and is usually due to incomplete excision. Cutaneous and extracutaneous glomus tumors are biologically benign, and metastasis or malignant changes are extremely rare [1, 2, 4, 8]. The current case showed no malignant features such as marked cytologic atypia, increased mitotic activity, or infiltrated growth pattern. There was also no recurrence after complete surgical excision.

Compared to the well-known subungual glomus tumor, extradigital glomus tumors may have atypical features, and the absence of objective findings often leads to a delay in diagnosis. A careful and comprehensive approach, including a thorough physical examination, comprehensive medical history, surveillance of the disease progress, and imaging studies, is essential upon clinical suspicion.

Although glomus tumors of the forearm are rare, an orthopedic surgeon should be aware of glomus tumors in the differential diagnosis of intravascular lesions of the upper limb. This tiny mass can cause severe pain to the patient, and a long history may unfortunately result in an improper diagnosis such as neuralgia, arthritis, and psychosomatic pain. Clinical suspicion plays an important role in management of this lesion, and only early surgical excision alleviates the patient's pain.
